# β_2_-Adrenoceptor Involved in Smoking-Induced Airway Mucus Hypersecretion through β-Arrestin-Dependent Signaling

**DOI:** 10.1371/journal.pone.0097788

**Published:** 2014-06-06

**Authors:** Yujiao Zhou, Yuan Zhang, Yang Guo, Youyi Zhang, Ming Xu, Bei He

**Affiliations:** 1 Department of Respiratory Medicine and Institute of Vascular Medicine, Peking University Third Hospital; Beijing Key Laboratory of Cardiovascular Receptors Research, Key Laboratory of Cardiovascular Molecular Biology and Regulatory Peptides, Beijing, People’s Republic of China; 2 Department of Respiratory Medicine, Changji Renmin Hospital, Changji, Xinjiang, People’s Republic of China; French National Centre for Scientific Research, France

## Abstract

Progression of chronic obstructive pulmonary disease is associated with small airway obstruction by accumulation of inflammatory mucous exudates. However, the mechanism of mucin hypersecretion after exposure to cigarette smoke (CS) is still not clear. In this study, we explored the contribution of β_2_-adrenoceptor (β_2_-AR) signaling to CS extract (CSE)-induced mucus hypersecretion *in vitro* and examined the effect of a β-blocker on airway mucin hypersecretion *in vivo.* NCI-H292 epithelial cell line was used to determine the contribution of β_2_-AR signaling to CSE-induced MUC5AC production by treatment with β_2_-AR antagonists propranolol and ICI118551 and β_2_-AR-targeted small interfering RNA. The effect of propranolol on airway mucus hypersecretion was examined in a rat model exposed to CS. MUC5AC expression was assayed by real-time PCR, immunohistochemistry and ELISA. β_2_-AR and its downstream signaling were detected by western blot analysis. We found that pretreating NCI-H292 cells with propranolol, ICI118551 for 30 min or β_2_AR–targeted siRNA for 48 h reduced MUC5AC mRNA and protein levels stimulated by CSE. However,inhibiting the classical β_2_AR–cAMP-PKA pathway didn’t attenuate CSE-induced MUC5AC production, while silencing β-arretin2 expression significantly decreased ERK and p38MAPK phosphorylation, thus reduced the CSE-stimulated MUC5AC production. In vivo, we found that administration of propranolol (25 mg kg^−1^d^−1^) for 28 days significantly attenuated the airway goblet cell metaplasia, mucus hypersecretion and MUC5AC expression of rats exposed to CS. From the study, β_2_-AR–β-arrestin2–ERK1/2 signaling was required for CS-induced airway MUC5AC expression. Chronic propranolol administration ameliorated airway mucus hypersecretion and MUC5AC expression in smoking rats. The exploration of these mechanisms may contribute to the optimization of β_2_-AR target therapy in chronic obstructive pulmonary disease.

## Introduction

The normal mammalian airway epithelium produces and is coated by mucins such as MUC5B and MUC5AC, which protect the airway epithelia against exogenous insult. However, excessive epithelial goblet cell metaplasia and mucus hypersecretion contribute to the pathology of many respiratory diseases such as chronic obstructive pulmonary disease (COPD), asthma, and cystic fibrosis. In COPD, mucin hypersecretion contributes to airway obstruction, accelerated decline of lung function and increased hospitalization and mortality [1]. MUC5AC is the predominant mucin produced from goblet cells in human airways; its expression is evoked by mediators such as neutrophil elastase, air pollutants and bacterial products [2], [3].

Cigarette smoking is the primary cause of COPD and induces the expression and production of MUC5AC *in vitro* and *in vivo* [4], [5]. As the first responders to cigarette smoke (CS), airway epithelial cells fulfill important roles: they recognize the inhaled CS components and mount a defense response but also produce various ligands that cause aberrant activation of some receptors such as epidermal growth factor receptor, which leads to epithelial cell hyperplasia and mucous cell metaplasia [6].

β_2_-adrenoceptor (β_2_-AR) is the most common adrenergic receptor subtype expressed in various types of lung cells, including airway epithelial cells. However, the role of β_2_-AR in airway mucus hypersecretion is still unknown. In an asthma murine model, the β-agonist formoterol inhibits airway goblet cell hyperplasia and MUC5AC protein expression, whereas administration of other β-agonists fails to improve mucus metaplasia [7], [8]. In addition, β_2_-AR signaling has been proven to be required for the full development of 3 cardinal features of asthma: mucous hypersecretion, airway hyperresponsiveness and the presence of inflammatory cells in the lungs and chronic administration of ICI118551 or nadolol reduced inflammation and mucous metaplasia, that may contribute to airflow obstruction and airway hyperresponsiveness of asthma[7], [9]. This evidence indicates β_2_-AR is involved in regulating lung inflammation and mucus secretion.

Cardiovascular disease is the most common co-morbidity of COPD because of the risk of smoking-induced atherosclerosis [10]. Prior studies have showed that use of β-blockers can reduce mortality and exacerbation when added to regular therapy for patients with COPD, independent of cardiovascular disease and cardiac therapy, without adverse effects on pulmonary function [11]. We previously showed that chronic exposure to propranolol, a nonselective β-AR antagonist, had a protective effect on airway smooth muscle contraction in rats exposed to CS [12]. These results suggest that β_2_-AR may be involved in the development of pathological changes associated with smoking. In the present study, we hypothesized that β_2_-AR signaling is required for airway MUC5AC expression and β-blocker treatment could improve CS-induced mucus hypersecretion by attenuating MUC5AC production. We explored the contribution of β_2_-AR and downstream signaling to MUC5AC expression in NCI-H292 cells, a mucin-expressing cell line from human airway epithelia, stimulated by CS extract (CSE) which is commonly used to mimic the effects of CS. We further examined the effect of chronic propranolol administration on MUC5AC production in rats exposed to CS.

## Materials and Methods

### Reagents

CSE, used to mimic the effects of CS, was purchased from Murty Pharmaceuticals (Lexington, KY, USA) and was prepared using a Phipps-Bird 20-channel smoking machine designed for FTC testing [13]. The particulate matter from Kentucky standard cigarettes (1R4F; approximately 11 mg of tar and 0.8 mg of nicotine/cigarette; University of Kentucky, KY, USA) was collected on Cambridge glass fibre filters and the amount obtained determined by weight increase of the filter. The average yield of CSE was 20.1 mg/cigarette. CSE was prepared by dissolving the collected smoke particulates in dimethyl sulfoxide (DMSO) to yield a 4% solution (w/v). The CSE was diluted into DMSO and aliquots were kept at −80°C. Propronolol (nonselective β-AR antagonist), ICI118551 (selective β_2_-AR antagonist), H89 (PKA inhibitor) PD98059 (ERK inhibitor) and SB203580 (p38MAPKK inhibitor) were from Sigma-Aldrich (St. Louis, MO, USA). Rp-8-Br-cAMPs and primary antibodies (Abs) for PKA, β-arrestin1, β-arrestin2, GAPDH and mouse or rabbit horseradish peroxidase-conjugated secondary Abs were from Santa Cruz Biotechnology (Santa Cruz, CA). Primary Abs for phospho-PKA, extracellular signal-regulated kinase 1/2 (ERK1/2) and phospho-ERK1/2 were from Cell Signaling Technology (CST, Boston, USA). Primary Abs for MUC5AC and β_2_-AR were from Abcam (London, UK).

### Cell Culture

NCI-H292 cells, a mucin-expressing cell line from human airway epithelia, were obtained from the Cell Resource Center (IBMS/CAMS/PUMC, China). Cells were maintained in DMEM supplemented with 10% fetal bovine serum, 1% penicillin/streptomycin (PS; 100 IU ml^−1^ each), at 37°C in a humidified 5% CO_2_ atmosphere and subcultured twice weekly. Confluent cells were incubated in serum-free DMEM for 12 h, then washed 3 times with 10% phosphate buffered saline (PBS), placed under serum-free conditions and exposed to the indicated concentration of CSE and inhibitors. Control cells were incubated with medium alone for the same period. After 24-h stimulation, samples of a cell-conditioned medium and/or cell lysates were collected by brief centrifugation and stored at −80°C. Most experiments, except for cell viability assays (96-well microplate), were involved 6-well plates or 35-mm dishes (Corning, USA) with 1×10^6^ cells/well.

### Silencing of β_2_-AR, β-arrestin1 and β-arrestin2 Expression

The two sets of siRNA oligos were synthesized by GenePharma Co. (Shanghai) as in Table 1. Target gene knockdown was achieved by transfection of NCI-H292 cells plated in 6-well dishes with doses of siRNA (100 pmol) and Lipofectamine2000 (Invitrogen, 5 µl) in 200 µl DMEM without serum; PS. A nonspecific siRNA was used for NC-siRNA. Lipofectamine2000 alone was a vehicle. For all siRNA transfections, 60% to 70% confluent monolayers were incubated with the transfection mixture in 1 ml serum-free medium for 6 to 8 h. Then serum was replenished and cells were used for various assays after 24 to 48 h.

**Table 1:** Si-RNA sequences used in the experiments.

Target Gene	Sequences (5′-3′)
Negative Control	sense -UUCUCCGAACGUGUCACGUTT
	Antisense -ACGUGACACGUUCGGAGAATT
ADRB2	sense -CGCCCAUAUUCUUAUGAAATT
	Antisense -UUUCAUAAGAAUAUGGGCGTT
ADRB2	sense -GCCAUUACUUCACCUUUCATT
	Antisense -UGAAAGGUGAAGUAAUGGCTT
ARRB1	sense -CGCCAGUAGAUACCAAUCUTT
	Antisense -AGAUUG GUAUCUACUGGCGTT
ARRB1	sense -GGAUCAUUGUUUCCUACAATT
	Antisense -UUGUAG GAAACAAUGAUCCTT
ARRB2	sense -GAUGAAGGAUGACGACUAUTT
	Antisense -AUAGUCGUCAUCCUUCAUCTT
ARRB2	sense -GCUCCACAUUCUGUAAGGUTT
	Antisense -ACCUUACAGAAUGUGGAGCTT

### Animal Experiment

**Figure 1:**
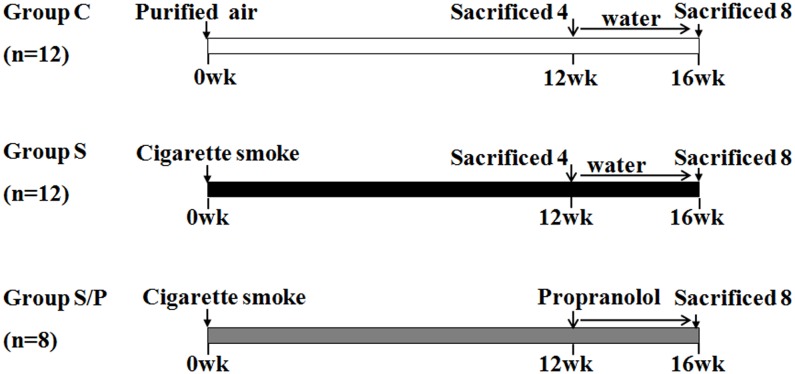
Study protocol. 32 rats were randomly divided into 3 groups for treatment: Group C (n = 12): exposure to air and administrated with distilled water as vehicle, Group S (n = 12): exposure to cigarette smoke and administrated with vehicle, Group S/P (n = 8) exposure to cigarette smoke and administrated with prapronolol. Rats in Group S and Group S/P were exposed to cigarette smoke by a whole-body inhalation instrument generated from commercial cigarettes at 20 cigarettes’ inhalation for 2 h followed by a 4-h recovery, repeated twice a day. Rats were exposed to CS 6 days per week. At 12 weeks later, 4 rats in Groups C and S were killed to evaluate lung function and histopathological alteration, respectively. From weeks 13 to 16, distilled water, 3–5 ml kg^−1 ^d^−1^, was intragastrically administered to Group C, Group S as vehicle, and propranolol, 25 mg kg^−1 ^d^−1^ (Kangpu Pharmaceutical Co., China) to Group S/P. The remaining rats were killed after 16 weeks of CS exposure.

This study was carried out in strict accordance with the recommendations in the Guide for the Care and Use of Laboratory Animals of the US National Institutes of Health. The protocol was approved by the Committee on the Ethics of Animal Experiments of Peking University (permit No.: LA2010-004). We housed 32 male Sprague-Dawley rats (7 weeks old) in divided cages (4 each) under specific pathogen–free conditions of the Animal Center of Peking University Health Science Center, Beijing. Animals were raised at room temperature with a natural light-dark cycle and had free access to food and water. All rats were anaesthetized by 20% uratan (0.5 mg/ml intra-peritoneal) for surgery, overdose of uratan for euthanasia, and all efforts including comfortable bedding, improved surgical intubation and standard surgical procedure, were made to minimize suffering. From weeks 1–16, rats in Group S and Group S/P were exposed to whole-body cigarette smoke in inhalation chambers (HOPE, China) generated from commercial cigarettes (Tar = 10 mg, cotinine = 0.9 mg, CO = 12 mg per cigarette) at 20 cigarettes’ inhalation for 2 h followed by a 4-h recovery, repeated twice a day. Rats were exposed to CS 6 days per week. The study protocol was showed in details as Figure 1.

### Lung Function Test

Rats were anesthetized by intraperitoneal injection of 20% urethane (1 mg kg^−1^), then respiration was maintained by a computer-controlled small-animal ventilator connected to the rat through a tracheal cannula. Respiratory function was measured as described [14]. Peak inspiratory flow, peak expiratory flow, intra-pressure (IP) and maximum rising slope of IP (IP slope) were analyzed by use of Chart 4.1 (AD Instruments, Australia).

### Bronchoalveolar Lavage (BAL) and Tissue Extraction

The left lobe and right posterior lobe of the lung were clamped at the hilus of the lung. In total, 1 ml PBS was infused and drawn back through the tracheal cannula 3 times. Total leukocytes in BAL fluid (BALF) were counted by use of a Coulter counter. The remaining BALF was centrifuged (3000 rpm, 10 min) and supernatants were stored at −80°C. The left lobe of the lung was preserved in 4% paraformaldehyde for histology. Tracheal samples 2–4 mm in diameter excised from the trachea were preserved in 3% glutaric dialdehyde for scanning electron microscopy and transmission electron microscopy. The right posterior lobe of the lung was preserved in liquid nitrogen for mRNA and protein analysis.

### Histopathology

Tissues were embedded in paraffin and stained with hematoxylin and eosin or periodic acid-Schiff (PAS). Images were acquired before any measurements and analyzed by a pathologist blinded to treatment. Pathological changes in small airways (≤2 mm in diameter) were assessed by inflammation scores as described [15]. The maximal luminal area and luminal content were calculated from images of 5 fields in airways ≤2 mm in diameter that were cross-sectioned, captured and viewed under a DM2500 optical microscope (LEICA, Germany). The content of mucin in the airway epithelium was measured by use of Image-Pro plus 5.0 as described [16].

### Electron Microscopy

Tracheal samples 2–4 mm in diameter were excised from the right principal bronchus of 3 rats under urethane anesthesia, taking care to avoid trachea cannula damage. Samples of untreated control mucosa were immediately immersed in the appropriate fixative as described and sent to the cytology and pathology labs of Peking University Health Science Center for conventional electron microscopy examination. Specimens were coated with gold-palladium in a Hummer II sputter-coating apparatus and examined under a JEOL JSM-5600LV scanning electron microscope. For transmission electron microscopy, mucosal slices were examined under JEOL JEM-1400 electron microscopes.

### Immunohistochemistry

After paraformaldehyde fixation, paraffinized 5-µm sections of lung tissue underwent immunohistochemical staining. For MUC5AC, the primary antibody was mouse polyclonal anti-MUC5AC (45M1; 1∶200 dilutions). After incubation at 4°C overnight, the biotinylated secondary goat anti-mouse antibody was applied (1∶500) for 30 min at room temperature and DAB chromogen was applied for 1 min.

### Real-time Quantitative PCR

Total RNA was extracted from frozen lung tissues or cultured NCI-H292 cells by the Trizol method (Takara Bio Inc. Japan). Real-time PCR involved use of Lightcycler480 (Eppendorf AG, Germany) with SYBR Green assay after reverse-transcribing 1 µg RNA by use of the Reverse Transcription System (Promega, USA). All data were normalized to mRNA levels of GAPDH. The primer pairs and expected lengths are in Table 2. The cycling conditions were 95°C for 30 s, then 40 cycles of 95°C for 5 s, 55°C for 30 s, and 65°C for 15 s. Relative gene expression was calculated as 2^−ΔΔCT^ [17]. When necessary, 0.8% agarose gel was used to separate the RT-PCR products.

**Table 2:** Primer sequences used in experiments.

Gene Name	Primer Sequences (5′-3′)	Product length (bp)
ADRB2-Homo	Forward-TCGTCATGTCTCTCATCGTCUTT	
	Reverse-AATGGCATAGGCTTGGTTCGATT	491
MUC5AC-Homo	Forward-ACCAATGCTCTGTATCCTTCCC	
	Reverse-TGGTGGACGGACAGTCACT	259
ARRB1-Homo	Forward-AAAGGGACCCGAGTGTTCAAG	
	Reverse-CGTCACATAGACTCTCCGCT	159
ARRB2-Homo	Forward-TCCATGCTCCGTCACACTG	
	Reverse-ACAGAAGGCTCGAATCTCAAAG	82
GAPDH-Homo	Forward-TGTGGGCATCAATGGATTTGGATT	
	Reverse-ACACCATGTATTCCGGGTCAATCTT	116
MUC5AC-Rattus	Forward-CTCCGTCTTAGTCAATAACCACC	
	Reverse-GGAACTCGTTGGATTTTGGACTG	221
GAPDH-Rattus	Forward-CCTCAAGATTGTCAGCAAT	
	Reverse-CCATCCACAG TCTTCTGAGT	103

### MUC5AC Mucin Assay

At 24 h after NCI-H292 cells were stimulated with CSE, cell culture media was collected for determining MUC5AC concentrations by use of an ELISA kit (R&D, USA). Total mucin and MUC5AC level in BALF was assayed by use of rat mucin and MUC5AC ELISA kits (4A Biotech Co., Beijing, China).

### Western Blot Analysis

Cells were harvested and lysed with lysis buffer (1% deoxycholic acid, 0.01 M Na_4_P_2_O_7_, 10% glycerol, 0.1 M NaCl, 5 mM EDTA, pH 8.0, 0.1% SDS, 0.02 M Tris-HCl, pH 7.4, 0.05 NaF, 1 mM Na_3_VO_4_, 100 µg ml^−1^ phenylmethylsulfonyl fluoride, and 10 µg/ml aprotinin). Cell lysates underwent protein determination by use of a Pierce BSA protein assay kit (Pierce, Rockford, IL, USA). Proteins (50 µg) were loaded onto 10% SDS polyacrylamide gel and electrophoretically transferred to nitrocellulose membrane (Pall, Port Washington, USA) that was incubated with antibodies according to the manufacturer’s protocol; immunolabelled bands were visualized by use of the SuperSignal West Pico chemiluminescence kit (Thermo, USA). Autoradiographs were quantified by densitometry by use of Image J software. Bands were normalized to GAPDH expression.

### Statistical Analysis

Data are presented as mean ± SEM. One-way ANOVA and Newman-Keuls multiple comparison test were used for normally distributed data as appropriate. A t-test was used to compare two different groups of treatment to each other. For multiple comparisons, two-way ANOVA was used. *P*<0.05 was considered statistically significant. Statistical calculations were carried out using Graph Pad Prism 5.0 (San Diego California, USA).

## Results

### β_2_-AR was Required for CSE-induced MUC5AC Production from NCI-H292 Cells

**Figure 2:**
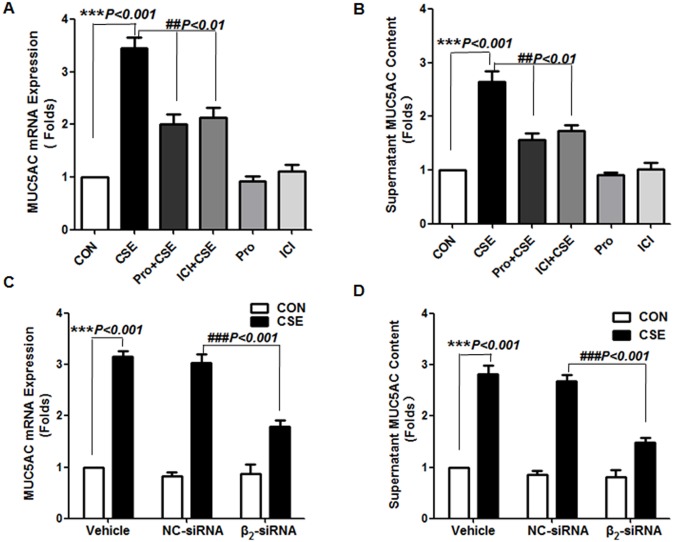
β_2_-AR blockade attenuates cigarette smoke extract (CSE)-induced MUC5AC production from NCI-H292 cells. Cells were pre-incubated with 10^−5^ M propranolol or 10^−6^ M ICI118551 for 30 min before adding CSE (Pro+CSE, ICI+CSE) or were incubated with media (CON), CSE, 10^−5^ M propranolol (Pro), or 10^−6^ M ICI118551 (ICI) alone for 24 h. Quantitative RT-PCR and ELISA of MUC5AC mRNA (A) and protein level (B), respectively, and MUC5AC mRNA (C) and protein secretion (D) of cells transfected with Lipofectamine2000 (vehicle), a nonspecific control siRNA (NC-siRNA, 100 nM) or β_2_-AR–targeted siRNA (β_2_-siRNA, 100 nM) for 48 h before incubation with or without CSE for 24 h. Data are means ± SEM (n = 3).

We preincubated NCI-H292 cells with the β_2_-AR antagonists propranolol or ICI118551 with or without CSE stimulation to explore the contribution of β_2_-AR to the CS-induced airway MUC5AC production. CSE at 0.1 mg ml^−1^ increased MUC5AC mRNA level and protein secretion in cells, and preincubation with antagonists reversed the increase (Figure 2). We further used two sets of β_2_-AR–targeted siRNAs to silence β_2_-AR expression on NCI-H292 cells, then incubated cells with CSE. β_2_-AR siRNA treatment reduced CSE-simulated MUC5AC mRNA and protein secretion in cells. Thus, β_2_-AR was required for the CSE-induced MUC5AC production from NCI-H292 cells. The indicated concentration of CSE was determined by a time- and dose-dependent pre-experiment (File ***S1***) and the β_2_-AR siRNA transfection and knockdown efficiency is shown in File ***S2***.

### CSE-induced MUC5AC Expression in NCI-H292 Cells was Independent of β_2_AR–cAMP–PKA

**Figure 3:**
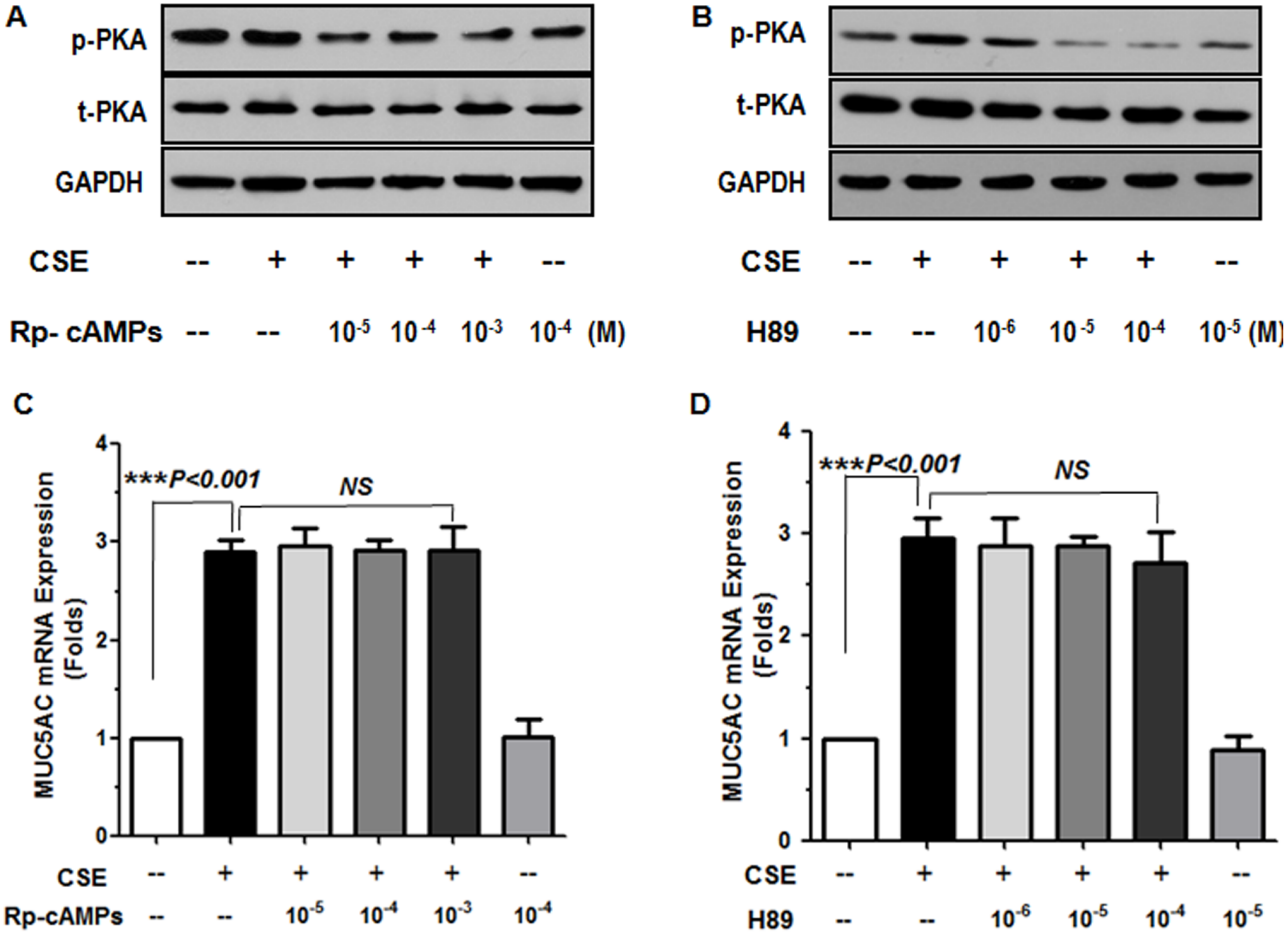
Effect of cAMP–protein kinase A (PKA) inhibition on CSE-stimulated MUC5AC expression. Western blot analysis of phospho-PKA (p-PKA) and total PKA (t-PKA) levels in cells preincubated with (A) Rp-8-Br-cAMPs or (B) H89 for 30 min before stimulation with or without CSE for 15 min. (C, D) RT-PCR analysis of MUC5AC mRNA expression after incubation with or without CSE for 24 h. Data are means ± SEM from 3 separate experiments. ***P<0.001 compared to CSE alone. NS, not significant.

β_2_-AR activation normally leads to increased intracellular cAMP levels, and cAMP production normally leads to activation of the catalytic subunit of PKA, which in turn phosphorylates cAMP-responsive element binding protein (CREB), allowing it to bind to CRE sites[18]. To determine whether CSE-induced MUC5AC expression in NCI-H292 cells operated through a β_2_AR–cAMP–PKA pathway, we pretreated cells with the cAMP analogue Rp-8-Br-cAMPs and PKA inhibitor H89. Inhibiting the cAMP–PKA pathway did not decrease the CSE-induced MUC5AC production (Figure 3). Therefore, CSE-induced MUC5AC expression was β_2_AR–cAMP–PKA independent.

### β_2_AR–β-arrestin2–ERK/p38MAPK Mediated CSE-induced MUC5AC Expression in NCI-H292 Cells

**Figure 4:**
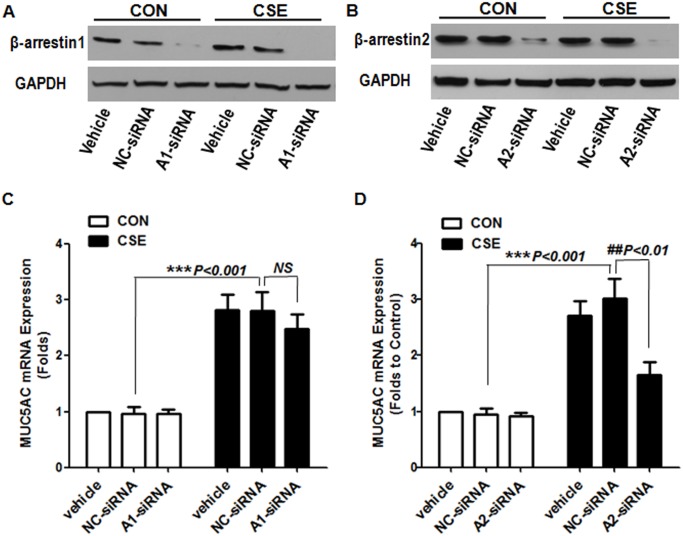
Effect of β-arrestin knockdown on CSE-stimulated MUC5AC expression. Cells were transfected with Lipofectamine2000 alone (vehicle), a nonspecific control siRNA (NC-siRNA, 100 nM), β-arrestin1–targeted siRNA (A1-siRNA, 100 nM) or β-arrestin2–targeted siRNA (A2-siRNA, 100 nM) for 48 h before incubation with or without CSE for 24 h. (A, B) Western blot analysis of β-arrestin1 and β-arrestin2 protein level to access the knockdown efficiency. (C, D) Quantitative RT-PCR analysis of MUC5AC mRNA expression. Data are means ± SEM (n = 3). NS, not significant.

β_2_-AR is a well-known G protein–coupled receptor (GPCR) and has many potential sites of phosphorylation. β-arrestin can uncouple the receptor from G protein, thus terminating desensitization and internalization of the receptor [19], [20]. In addition, β-arrestin can serve as multi-functional adaptors and signal transducers, directing the recruitment, activation, and scaffolding of cytoplasmic signaling molecules, including mitogen-activated protein kinase (MAPK) and the Ser-Thr kinase Akt [21], [22]. MUC5AC gene expression is critically regulated by MAPK (ERK, p38MAPK and JNK) [23], [24]. We sought to determine whether β_2_-AR activation induced airway epithelial MUC5AC production by CSE via a β-arrestin–MAPK pathway. We pretreated cells with 2 sets of β-arrestin-targeted siRNA, β-arrestin1 siRNA and β-arrestin2 siRNA. CSE-stimulated MUC5AC expression was significantly decreased in cells pretreated with β-arrestin2 siRNA but not β-arrestin1 siRNA (Figure 4).

**Figure 5:**
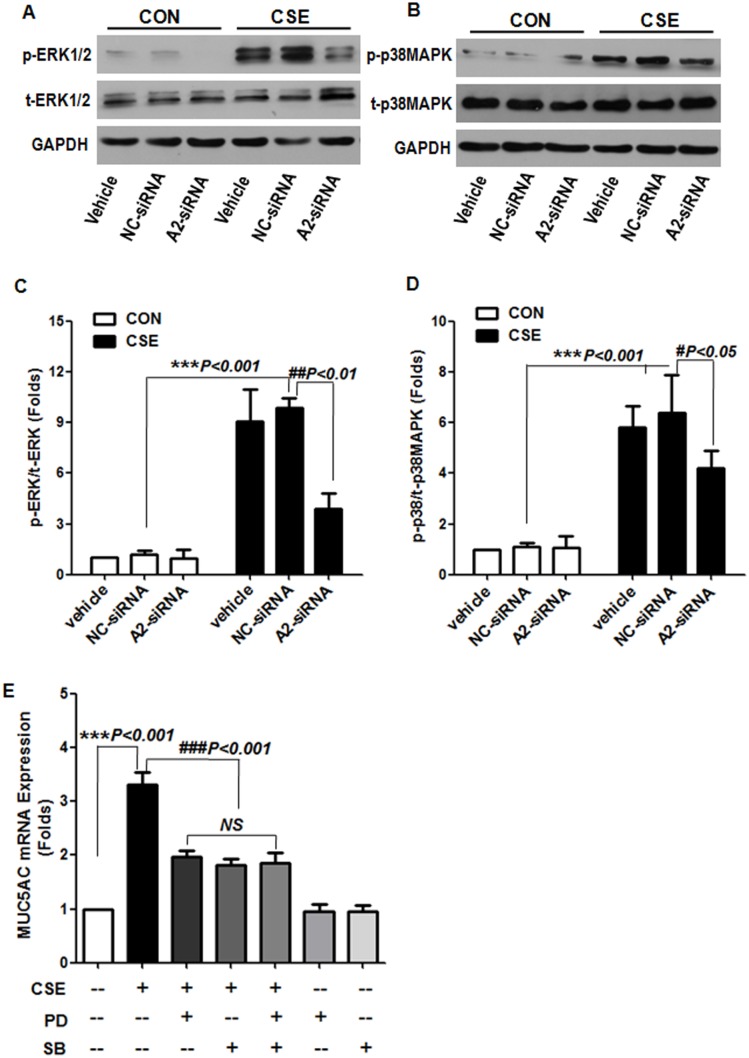
Effect of β-arrestin knockdown on CSE-stimulated MAPK activation. Western blot analysis of phospho-ERK1/2 (p-ERK1/2), total ERK1/2 (t-ERK1/2), phospho-p38MAPK (p- p38MAPK) and total p38MAPK (t-p38MAPK) in cells treated with Lipofectamine2000 (vehicle), nonspecific control siRNA (NC-siRNA, 100 nM) or β-arrestin2–targeted siRNA (A2-siRNA, 100 nM) before CSE stimulation as in Figure 4. (A, B) Representative western blot is from 3 independent experiments. (C, D) Data are the means ± SEM of 3 separate experiments. Cells were pretreated with ERK1/2 inhibitor PD98059 (50 µM) or p38MAPK inhibitor (5 µM) for 60 min before CSE treatment. (E) Quantitative RT-PCR analysis of MUC5AC mRNA expression. Data are means ± SEM (n = 3). NS, not significant.

Because β-arrestin2 is associated with MAPK signaling [25], we observed intracellular ERK1/2 and p38MAPK activation after β-arrestin2 siRNA pretreatment. We further pretreated cells with the ERK inhibitor PD98059 or p38MAPK inhibitor SB203580 and examined MUC5AC expression. Consistently, ERK1/2 and p38MAPK phosphorylation was significantly suppressed in cells pretreated with β-arrestin2 siRNA, and cultures pretreated with PD98059 or SB203580 showed decreased CSE-induced MUC5AC production, but their combination failed to further reduce the MUC5AC production (Figure 5). Therefore, β-arrestin2**–**ERK1/2 and p38MAPK is required for β_2_-AR mediated MUC5AC expression induced by CSE.

Much clinical evidence has shown that β-blockers are safe and beneficial for patients with COPD and cardiovascular disease. However, we need solid data before recommending regular use of β-blockers to reduce airway or lung inflammation and mucus hypersecretion in COPD, because of concerns that β-blockers might induce bronchospasm and worsen lung function [26], [27]. However, these concerns have been challenged by recent evidence: the risk of bronchospasm can be reduced by starting β-blockers at a lower dose and slowly titrating up [28]. Therefore, we used long-term and low-dose propranolol as a “conservative treatment” and observed its effect on lung function, airway/lung inflammation and mucus hypersecretion in rats exposed to CS to verify the findings of β_2_-AR *in vitro*. The weight change as well as the survival rate of rats during the study was showed in ***File S3***.

### Chronic Propranolol Administration Attenuated Airway Mucus Hypersecretion in Rats Exposed to CS

**Figure 6:**
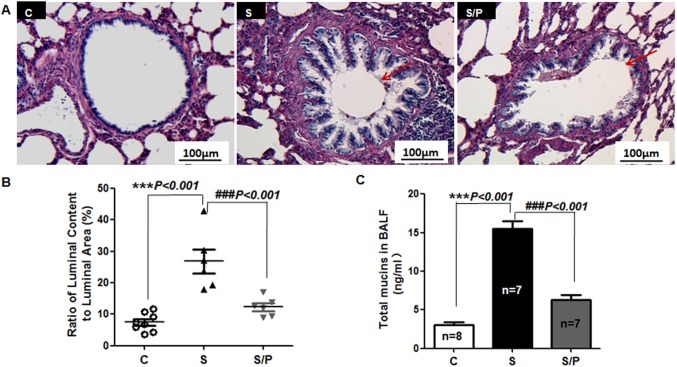
Chronic administration of propranolol attenuates airway mucus obstruction in rats exposed to CS. (A) Representative rat lung sections stained with periodic acid-Schiff exposed to air and vehicle (Group C), CS-exposed rats with vehicle (Group S) or prapronolol for 4 weeks (Group S/P). Scale bar, 200 µm. (B) Quantification of the area of airway lumen and luminal content of airways ≤2 mm in diameter. (C) ELISA of total mucins in bronchoalveolar fluid (BALF). Data are mean ± SEM. ***P<0.001, compared with Group C; ###*P*<0.001 compared with Group S.

**Figure 7:**
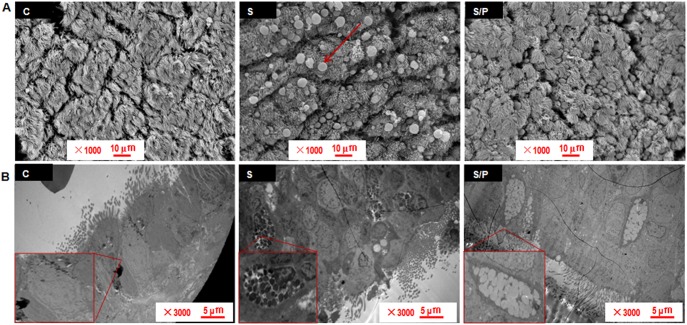
Chronic administration of propranolol inhibits airway goblet cell metaplasia and mucin hypersecretionin in rats exposed to CS. (A) Scanning electron microscopy of the luminal surface shows the apices of ciliated and goblet cells (arrow). Bar = 10 µm. (B) Transmission electron microscopy of airway epithelia shows massive mucin-containing secretory granules in goblet cells in response to CS. Bar = 5 µm.

We first evaluated the general histopathological changes and lung function in pure air-exposed rats, CS-exposed rats and CS-exposed rats administrated propranolol. Chronic propranolol administration ameliorated a number of CS-induced pathological alterations such as airway occlusion, fibrosis, and smooth muscle proliferation, especially goblet cell metaplasia (***File S4***). Among the 4 lung function indexes, propranolol decreased the peak expiratory flow, intra-airway pressure and intra-airway pressure slope (***File S5***). As compared with air-exposed rats, CS-exposed rats showed increased airway mucus, and propranolol treatment significantly decreased airway luminal mucus accumulation and total mucins in BALF (Figure 6). Mucins secreted from goblet cells are the primary components maintaining mucus viscosity. In rats exposed to CS, airways showed massive goblet cell metaplasia, mucus hyperplasia and collapsed cilia between the dense, overlying mucus and the epithelial cell surface; treatment with propranolol significantly decreased the production of secretory granules induced by CS (Figure 7).

### Chronic Propranolol Administration Decreased MUC5AC Expression in Lungs of Rats Exposed to CS

**Figure 8:**
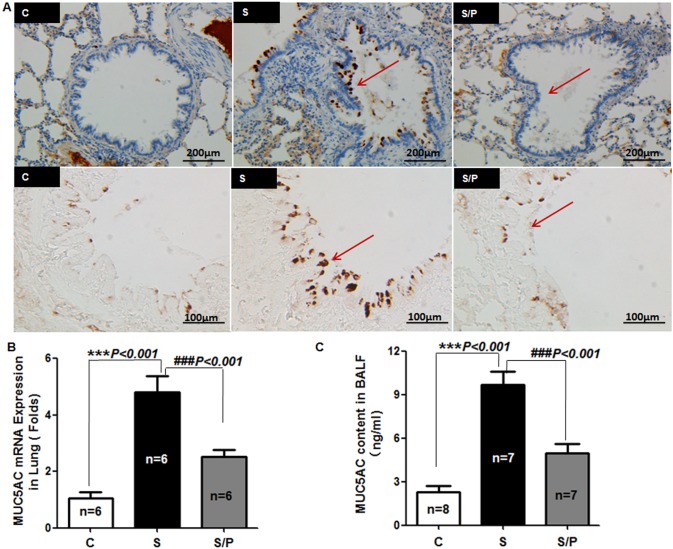
Chronic administration of propranolol decreases airway epithelial MUC5AC expression in rats exposed to CS. (A) MUC5AC glycoprotein expression in lung tissue by immunohistochemical staining. Bar = 200 µm and 100 µm in the upper and lower images, respectively. (B) Quantitative RT-PCR analysis of mRNA level of MUC5AC in lung homogenate. (C) ELISA of MUC5AC protein in BALF. Data are means ± SEM in each group.

**Figure 9:**
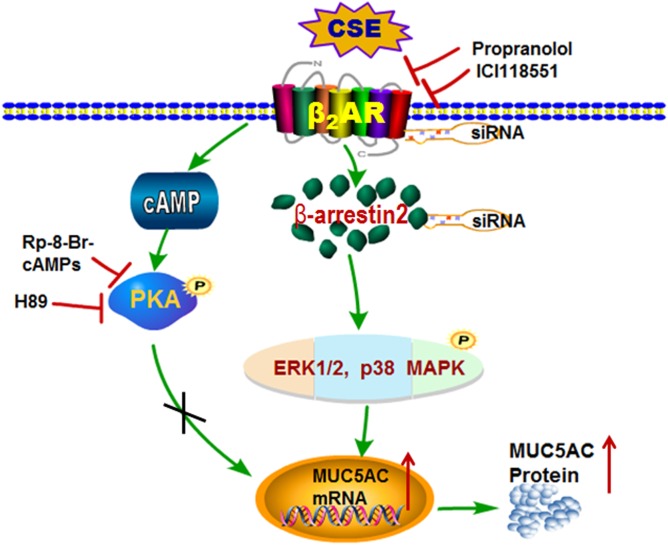
Schematic of the β_2_-AR signaling pathway involved in CS-induced upregulation of MUC5AC production in NCI-H292 cells, a mucin-expressin cell line from human airway epithelia. Wtih CSE stimulation, *β_2_-AR* is activated, which increases cAMP and recruits many *β*-arrestins. Increased cAMP binds and activates PKA but does not stimulate MUC5AC transcription and production. *β*-arrestin2 phosphorylates ERK1/2 and p38MAPK, the key players, which activate transcription factors to stimulate MUC5AC transcription and production. β-AR antagonists, kinase inhibitors and molecular biological methods of interfering with signaling molecules are also shown.

MUC5AC is the main component of airway mucus. We further examined the effect of propranolol on MUC5AC expression and secretion. Chronic administration with low-dose propranolol significantly decreased CS-stimulated MUC5AC expression in airway epithelia, MUC5AC mRNA level in lung homogenates and protein level in BALF (Figure 8). Thus, β-AR was involved in regulating the airway MUC5AC production. The detailed mechanism of β_2_-AR involved in cigarette smoke -induced MUC5AC production was shown in Figure 9.

## Discussion

Cigarette smoking is the primary risk factor of COPD -associated with airway mucus hypersecretion. MUC5AC is the major inducible mucus associated with COPD progression [29]. We found that blocking β_2_-AR with propranolol, ICI118551 or siRNA significantly decreased CSE-induced MUC5AC expression in NCI-H292 cells and chronic administration of propranolol, a nonselective β-AR antagonist, significantly decreased the airway epithelial mucus hyperproduction in rats exposed to CS. These effects may depend inhibition of CS/CSE-simulated β_2_-AR–β-arrestin**–**ERK1/2 and p38MAPK phosphorylation.

β_2_-AR signaling shows tissue specificity and diverse bioeffects. A ligand may simultaneously activate more than one G protein–coupled receptor signaling pathway and certain ligands may be able to preferentially direct receptor signaling to a specific pathway. In airway smooth muscle cells, cAMP–PKA–CREB is considered the conventional β_2_-AR–activated signal pathway, which mainly causes bronchodilatation. In macrophages, β_2_-AR activation has anti-inflammatory properties, inhibiting NF-κB activation and cytokine production induced by pro-inflammatory stimuli while increasing IL-1β and IL-6 protein production in the absence of pro-inflammatory stimuli [30]. Collectively, these findings suggest that β_2_-AR produces different signals in different stimuli and the signals may have a different role in different cells. Our study of ICI118551, a selective β_2_-AR antagonist, and β_2_-AR–targeted siRNA administered to NCI-H292 cells, a cell line from human airway epithelia, revealed that β_2_-AR participated in MUC5AC production induced by CSE. β_2_-AR blockade decreased the induction of respiratory MUC5AC mucin by CSE both at the mRNA and protein levels (Figure 2).

We first observed the effect of the conventional β_2_-AR–cAMP–PKA pathway on MUC5AC production; however, the results suggested that CSE-stimulated MUC5AC production was independent of PKA (Figure 3). Our results suggested that the MUC5AC expression was dependent on MAPK activation, but independent of PKA activation, which agreed with study of the anti-inflammatory role of the cAMP–PKA pathway [31]. Rp-8-Br-cAMP is the most potent cAMP antagonists. Its role is to occupy cAMP binding sites and prevents dissociation and thus activation of the kinase holoenzyme. Rp-8-Br-cAMP discriminates between the two isozymes of protein kinase A and prefers type I [32]. Thus,different doses of Br-8-Rp-cAMPs application showed no dose-response of MUC5AC expression, which indicated that the MUC5AC expression was independent of PKA activation. A similar result was showed after pretreating cells with PKA inhibitor H89. Other studies have shown that long-term tobacco smoke resulted in desensitization of the cough receptors within the airway epithelium and internalization of toll-like receptor 4 on human macrophages and cystic fibrosis transmembrane conductance regulator on pulmonary epithelia [33], [34], [35].

As mentioned previously, β-arrestin could desensitize and internalize activated β_2_-AR [36], [37]. Moreover, β-arrestin, as non-classical β_2_-AR–dependent signal binding to the endocytic machinery, use additional docking sites to link various MAPKs. ERK1/2, p38-MAPK and JNK are the main members of the MAPK family involved in regulating MUC5AC expression [38]. Mucus overproduction induced by smoke inhalation was found associated with increased epithelial MUC5AC protein expression and depended on activation of the JNK pathway [39]. A second PKA-independent and MAPK-dependent pathway of β_2_-AR activation has been reported in neurons [40].

β-arrestin has 2 isoforms, β-arrestin1 and β-arrestin 2. We used siRNA to silence the expression of β-arrestin-1 and β-arrrestin-2 in NCI-H292 cells. Silencing β-arrrestin2 expression suppressed CSE-stimulated ERK1/2 activation, thus inhibiting MUC5AC production (Figures 4 and 5). CSE exposure may have induced β_2_-AR internalization, because both isoforms of β-arrestin can promote desensitization of β_2_-AR, but only β-arrestin2 promotes internalization of β_2_-AR [37], [41]. We demonstrated a potent stimulatory effect of CSE on MUC5AC mucin secretion from NCI-H292 cells, and activation of β_2_-AR with β-arrestin2 but not PKA was involved in MUC5AC gene expression. We proposed a detailed mechanism of β_2_-AR involved in CSE-induced MUC5AC production: wtih CSE stimulation, β_2_-AR is activated, which increases cAMP and recruits many β-arrestins. Increased cAMP binds and activates PKA but does not stimulate MUC5AC transcription and production. β-arrestin2 phosphorylates ERK1/2 and p38MAPK, the key players, which activate transcription factors to stimulate MUC5AC transcription and production (Figure 9).

We further established a rat model with a prominent feature of airway inflammation and mucus secretion by CS exposure and evaluated after chronic low-dose propranolol intervention. Propranolol, one of non-selective β-blockers, is the most commonly prescribed classes of cardiovascular medication. Despite increasing evidence that BBs are safe and can actually be beneficial in patients with COPD, their use in this population is limited [42]. Because of concerns that BBs might induce bronchospasm and worsen lung function, there has been uncertainty with regard to using β-blockers in COPD patients. Such concerns, however, have been challenged by recent evidence: cardio-selective β-blockers are less likely to cause bronchospasm, and, additionally, the risk of bronchospasm can be reduced by starting β-blocker treatment at a lower dose and slowly titrating up [28], [43]. Moreover, treatment with BBs may reduce the risk of exacerbations and improve survival in patients with COPD but without overt cardiovascular disease [44]. In this study, echocardiography was performed on the rats at baseline and repeated after smoking for 4 months to evaluate the function of heart, but no overt cardiovascular disease was found in the rat model (data not shown). Comparatively, goblet cell hyperplasia and mucin hypersecretion occurred in airway epithelia with CS exposure and could be inhibited by chronic administration of propranolol (Figures 7 and 8). This result suggested that the benefit from using propranolol in CS induced animals did not necessarily correlate with reduced cardiovascular side effects. The possible explanations for this result include 1) propranolol inhibiting the activation of the sympathetic nerve. Our previous study showed elevated serum levels of norepinephrine in CS-exposed rats, and propranolol significantly decreased the serum norepinephrine levels [45]. However, in airways of all species studied, particularly humans, cholinergic mechanisms predominate and the sympathetic nervous system influences the volume secretion and mucus content probably by activation of alpha-adrenergic pathways [46]. 2) Propranolol increases β_2_-AR level in airway epithelia. In a murine asthma model, chronic administration of β-blockers attenuating mucus production was associated with upregulated β_2_-AR level in airway epithelia, and transgenic overexpression of β_2_-AR in airway epithelial cells decreased bronchoconstriction and airway hyperresponsiveness [7], [47]. However, we did not find a significant altered β_2_-AR level in airway epithelia after propranolol treatment (data not shown). 3) Propranolol inhibits β_2_-AR downstream signaling molecules. β_2_-AR signaling was found required for the full development of mucous metaplasia, and inhibition of all β_2_-AR signaling but not biased agonism was responsible for the beneficial effects of chronic inverse-agonist treatment on mucous metaplasia [9]. Moreover, β_2_-AR is the most common β-AR subtype expressed in various cell types in the lung including airway epithelial cells. β_2_-AR level has been found increasing along the human airway, and the density in the subsegmental bronchus and lung parenchyma was approximately two-fold higher than that of muscarinic acetylcholine receptors in the same region. β_1_-AR was also detected in lung parenchyma but not in the bronchus, which suggests the inhibition was mainly mediated through β_2_-AR [48].

Our study contains some limitations. 1) Mainstream CS is a complex mixture inhaled into the respiratory system. Approximately 4,700 substances have been identified in fresh tobacco smoke. Many factors influence CS-stimulated phosphorylation of ERK1/2, such as epidermal growth factor receptor, toll-like receptor or NADPH oxidase, and interactions between these factors and β_2_-AR [49], [50]. 2) Propranolol is a nonselective β-AR antagonist, but the contribution of β_1_-AR to MUC5AC production is not involved here. However, our previous study found that treatment with metoprolol, a β_1_-AR selective antagonist, did not improve airway mucus hypersecretion in a rat model of CS exposure (data not shown). The protective effect of propranolol in the rat model exposed to CS may be mainly achieved by inhibiting β_2_-AR signals. 3) *In vitro* systems usually lack complex interactions with other cell types and the disease process can be affected by the complex cytokine milieu established by inflammatory cells in addition to CS exposure. Thus, the findings *in vitro* may not exactly reflect responses *in vivo*.

In conclusion, our findings indicate a detrimental effect of β_2_-AR activation on airway mucus hypersecretion. Some pathological signals of β_2_-AR are initiated under CS exposure and proper application of β-blockers may, to a point, benefit airway disease. The exploration of these mechanisms may contribute to the optimization of β_2_-AR target therapy in smoking associated airway disease, like COPD.

## Supporting Information

File S1 **Dose- and time-dependent effect of CS extract (CSE) on cell viability and MUC5AC production from NCI-H292 cells.**
(DOCX)

File S2 **Transfection and knockdown efficiency of β_2_-siRNAs.**
(DOCX)

File S3 **The body weight of rats in all 3 groups at different time.**
(DOCX)

File S4 **Effect of propranolol on pulmonary pathological parameters in rats exposed to cigarette smoke.**
(DOCX)

File S5 **Effect of propranolol on lung function.**
(DOCX)
