# Supplementary material for: β2-Adrenoceptor Involved in Smoking-Induced Airway Mucus Hypersecretion through β-Arrestin-Dependent Signaling
Source: PLoS One. 2014 Jun 6;9(6):e97788. doi: 10.1371/journal.pone.0097788 (PMC4048185; doi:10.1371/journal.pone.0097788)
Supplement: File S1 — Dose- and time-dependent effect of CS extract (CSE) on cell viability and MUC5AC production from NCI-H292 cells. (DOCX) [file pone.0097788.s001.docx]

**Dose- and time-dependent effect of CS extract (CSE) on cell viability and MUC5AC production from NCI-H292 cells**


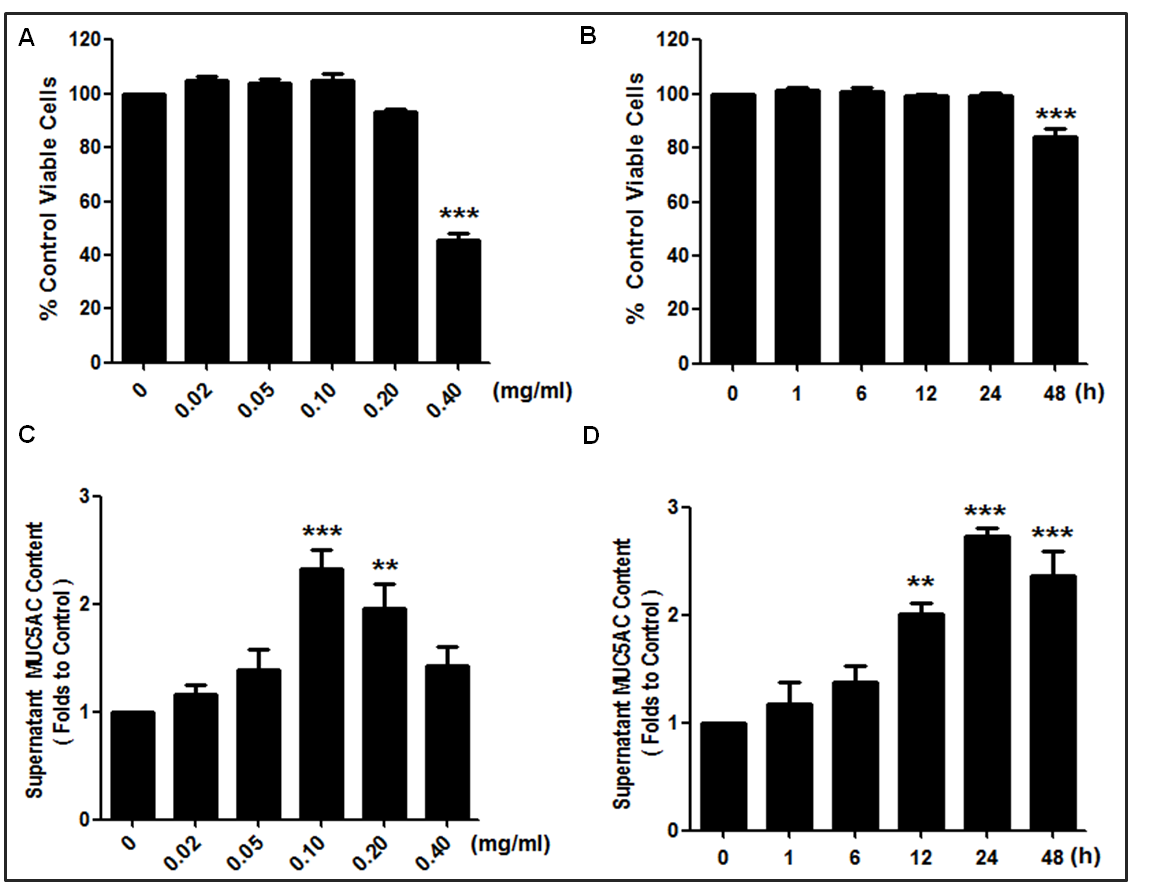


**Figure S1. Dose- and time-dependent effect of CS extract (CSE) on cell viability and MUC5AC production from NCI-H292 cells.**  The original concentration of cigarette smoke condensate was 40 mg ml^-1^ and the recommended maximum permissive concentration was 0.2 mg ml^-1^ according to the manufacturer. To explore the optimal concentration, cells were incubated in 96-well plates and stimulated for 24 h with different concentrations of CSE. Similarly, cells were stimulated with 0.1 mg ml^-1^ of CSE for the different stages. Cell viability was 90% and 35% stimulated with 0.2 mg ml^-1^ and 0.4 mg ml^-1^ of CSE for 24 h, respectively. Cell viability was more than 95% when stimulated with CSE less than 0.1 mg ml^-1^. At the same time, cells were incubated in 6-well plates determine the dose- and time- dependent effects of CSE on MUC5AC production from NCI-H292 cells. (A, B) Cell viability was with dose and time of CSE administration. (C, D) MUC5AC secretion was with dose and time of CSE administration. Data are mean± SEM from 3 independent experiments. ***P*<0.01, ****P*<0.001, compared to serum-free medium alone.
