# Supplementary material for: β2-Adrenoceptor Involved in Smoking-Induced Airway Mucus Hypersecretion through β-Arrestin-Dependent Signaling
Source: PLoS One. 2014 Jun 6;9(6):e97788. doi: 10.1371/journal.pone.0097788 (PMC4048185; doi:10.1371/journal.pone.0097788)
Supplement: File S2 — Transfection and knockdown efficiency of β2-siRNAs. (DOCX) [file pone.0097788.s002.docx]

**Transfection and knockdown efficiency** **of** β_2_-**siRNAs**

**
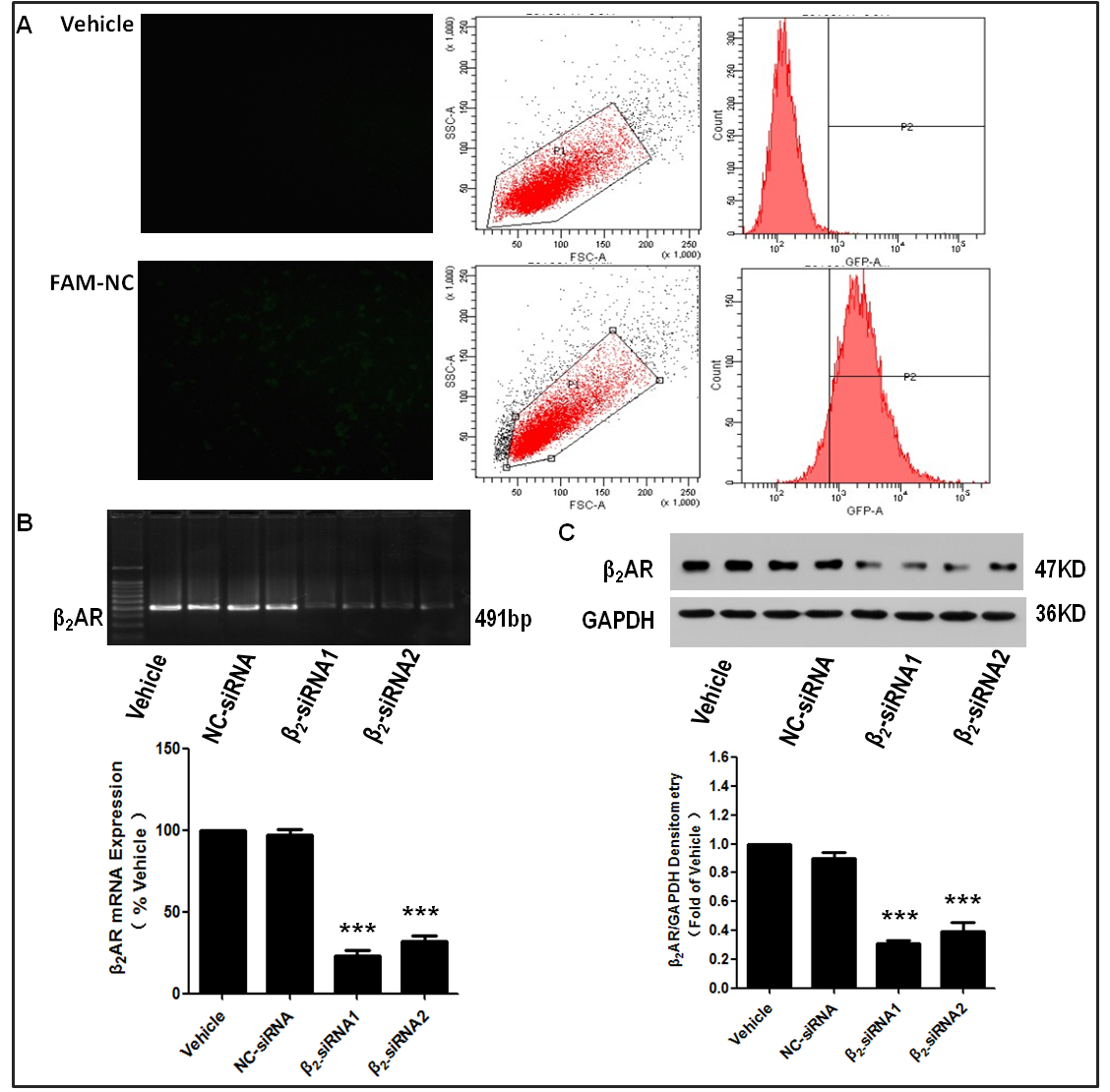
**

**Figure S2. Transfection and knockdown efficiency** **of** β_2_-**siRNAs** (A) Cells were transfected with a nonspecific, fluorescently tagged siRNA construct (FAM-NC) to assess transfection efficiency and viability by flow cytometry. (B, C) Quantitative RT-PCR and western blot analysis of mRNA and protein level of β_2_-AR in cells transfected with 2 sets of siRNA targeting β_2_-AR (β_2_-siRNA1, β_2_-siRNA1) or a nonspecific control oligo (NC-siRNA) at 100 nM for (B) 24 h and (C) 48 h. ***P<0.001 compared with NC-siRNA.
