# Supplementary material for: β2-Adrenoceptor Involved in Smoking-Induced Airway Mucus Hypersecretion through β-Arrestin-Dependent Signaling
Source: PLoS One. 2014 Jun 6;9(6):e97788. doi: 10.1371/journal.pone.0097788 (PMC4048185; doi:10.1371/journal.pone.0097788)
Supplement: File S3 — The body weight of rats in all 3 groups at different time. (DOCX) [file pone.0097788.s003.docx]

**General information of the rat model**

Four rats in Group C and Group S were killed respectively after 12weeks. During the experiment, one rat died in Group S and Group S/P, respectively. The body weight of rats in all three groups was assayed at baseline and repeated after smoking for 12 weeks and 16weeks. Compared with the body weight of rats in Group C at the same time, there was a significant decreased body weight after smoking for 12 weeks (*P<0.001*). Propranolol application did not affect the body weight.


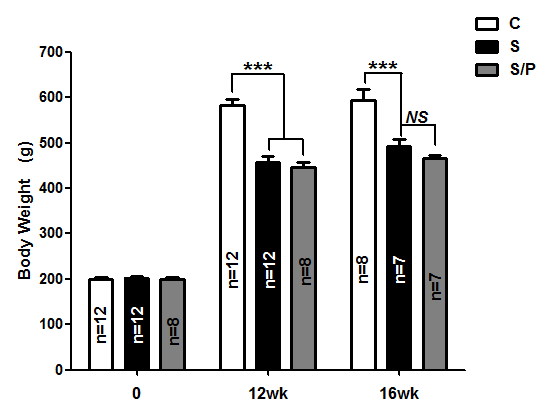


**Figure S3. The body weight of rats in all 3 groups at different time**

****P*<0.001, compared with Group C; NS, no significant difference.
