# Supplementary material for: β2-Adrenoceptor Involved in Smoking-Induced Airway Mucus Hypersecretion through β-Arrestin-Dependent Signaling
Source: PLoS One. 2014 Jun 6;9(6):e97788. doi: 10.1371/journal.pone.0097788 (PMC4048185; doi:10.1371/journal.pone.0097788)
Supplement: File S4 — Effect of propranolol on pulmonary pathological parameters in rats exposed to cigarette smoke. (DOCX) [file pone.0097788.s004.docx]

**Effect of propranolol on airway/pulmonary pathological score in rats exposed to cigarette smoke**

**
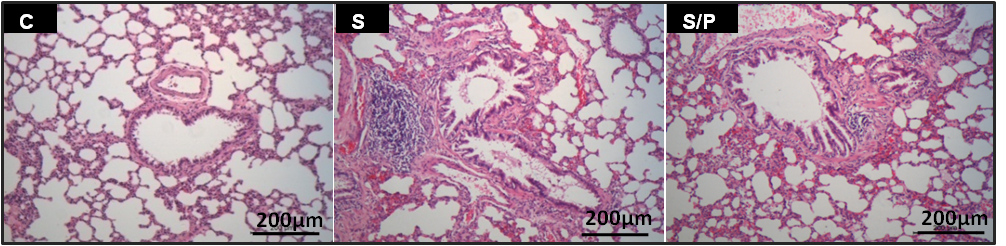
**

**Figure S4. Effect of propranolol on pulmonary pathological parameters in rats exposed to cigarette smoke** Hematoxylin and eosin staining of lung sections from rats in three groups. Bar=200 μm.

The pathology score was assigned by evaluation of 9 variables, including mean airway occlusion, epithelial necrosis erosion, goblet cell metaplasia, inflammatory cell infiltration, squamous metaplasia, fibrosis and smooth muscle proliferation, pigmentation and emphysema (Table S1). Evaluation of each variable was assigned a score ranging from 0, normal, and 1 to 4, mild to severe damage (1–25% to 75–100%). For each small airway, the scores for all the features were summed and expressed as a proportion of the maximum possible small airway disease score, all features thus being given the same weight in each airway. The separate 9 scores were summed as the total pathological score. Chronic administration of propranolol improved a number of cigarette smoke-induced pathological alterations such as airway occlusion, fibrosis, and smooth muscle proliferation etc, especially the improvement on goblet cell metaplasia (0.9±0.1 vs 1.7±0.3) and inflammatory cell infiltration (1.4±0.2 vs 2.3±0.2). However, propranolol treatment failed to improve the emphysema and epithelial necrosis.

**Table S1. Pathological score of rat lung tissue in 3 groups**

| Parameters | C (n=6) | S (n=6) | S/P (n=6) |
| --- | --- | --- | --- |
| Airway occlusion | 1.3±0.2 | 2.7±0.3** | 2.0±0.0^#^ |
| Epithelial necrosis erosion | 0 | 1.2±0.3*** | 0.7±0.2*^#^ |
| Goblet cell metaplasia | 0.3±0.2 | 1.7±0.3*** | 0.9±0.1^#^ |
| Squamous metaplasia | 0 | 0 | 0 |
| Inflammatory cell infiltration | 1.3±0.2 | 2.3±0.2** | 1.4±0.2^##^ |
| Fibrosis | 0 | 1.3±0.2*** | 0.9±0.1*^#^ |
| Smooth muscle proliferation | 0.1±0.1 | 2.1±0.3*** | 1.0±0.2*^##^ |
| Pigmentation | 0 | 2.7±0.2*** | 2.6±0.3*** |
| Emphysema | 0 | 1.3±0.2*** | 1.0±0.0** |
| Total score | 3.0±0.6 | 15.3±1.7*** | 10.6±0.5*^#^ |

Data are mean±SEM (n=6). **P*<0.05, ***P*<0.01, ****P*<0.001compared with Group C;

^#^*P*<0.05, ^##^*P*<0.01, compared with Group S.
