# Supplementary material for: β2-Adrenoceptor Involved in Smoking-Induced Airway Mucus Hypersecretion through β-Arrestin-Dependent Signaling
Source: PLoS One. 2014 Jun 6;9(6):e97788. doi: 10.1371/journal.pone.0097788 (PMC4048185; doi:10.1371/journal.pone.0097788)
Supplement: File S5 — Effect of propranolol on lung function. (DOCX) [file pone.0097788.s005.docx]

**Effect of propranolol on lung function in a cigarette smoke-induced rat model**

Pulmonary functions decreased in rats exposed to cigarette smoke. PEF was 33.3% lower in rats of Group S than in Group C, whereas IP slope was 49.7% higher (*P*<0.01). However, PEF in propranolol-treated rats was not lower than Group S, while IP slope was 29.9% lower than Group S, which suggested that administration of propranolol in rats exposed to cigarette smoke had no aggravation, even diminishment of lung function decline (Table S2).

**Table S2. Effect of propranolol on lung function**

| Group | PIF (ml/S) | PEF (ml/S) | IP (mmHg) | IP slope (mmHg/S) |
| --- | --- | --- | --- | --- |
| C(n=6) | 9.78±0.23 | 7.76±0.21 | 2.15±0.46 | 103.68±4.41 |
| S (n=4) | 10.05±0.14 | 5.82±0.30** | 2.63±0.76 | 155.14±4.86*** |
| S/P (n=7) | 9.66±0.33 | 6.51±0.36 | 2.04±0.61 | 108.72±7.27**^###^** |

PIF, peak inspiratory flow; PEF, peak expiratory flow; IP, intra-pressure; IP slope, and maximum rising slope of IP. Data are mean±SEM; ***P*<0.01, ****P*<0.001 compared with Group C; ^###^*P*<0.001 compared with Group S.
